# Predictive value of elevated alanine aminotransferase for in-hospital mortality in patients with acute myocardial infarction

**DOI:** 10.1186/s12872-021-01903-z

**Published:** 2021-02-09

**Authors:** Jian Li, Zhuo Zhao, Hui Jiang, Minjie Jiang, Ge Yu, Xu Li

**Affiliations:** 1 Department of Hepatology, The First Hospital of Jilin University, No. 71, Xinmin Street, Changchun, Jilin China; 2 Department of Cardiology, The Second Hospital of Jilin University, Changchun, Jilin China; 3 Medical Oncology Department, Jilin Cancer Hospital, Changchun, Jilin China

**Keywords:** Liver enzymes, Alanine transaminase, eGFR, Fasting plasma glucose, Acute myocardial infarction, Mortality

## Abstract

**Background and aims:**

Liver enzymes, including alanine aminotransferase (ALT) and aspartate aminotransferase (AST), are markers of hepatic dysfunction and fatty liver disease. Although ALT and AST have been suggested as risk factors for cardiovascular disease, their role as predictors of mortality after acute myocardial infarction (AMI) has not been established. The objective of this study was to investigate the predictive value of ALT and AST for mortality in patients with AMI.

**Methods:**

We analyzed records of 712 patients with AMI and no known liver disease treated at the Department of Cardiovascular Center in the First Hospital of Jilin University. The primary outcome was all-cause in-hospital mortality. Relationships between primary outcome and various risk factors, including serum transaminase levels, were assessed using multivariate logistic regression analysis.

**Results:**

Age (P < 0.001), hypertension (P = 0.034), prior myocardial infarction (P < 0.001), AST (P < 0.001), ALT (P < 0.001), creatinine (P = 0.007), blood urea nitrogen (P = 0.006), and troponin I (P < 0.001) differed significantly between ST-segment elevation myocardial infarction (STEMI) and non-STEMI. The following factors were associated with an increased risk of in-hospital all-cause mortality in patients with AMI: ALT ≥ 2ULN (adjusted odds ratio [AOR] 2.240 [95% confidence interval (CI), 1.331–3.771]; P = 0.002); age ≥ 65 year (AOR 4.320 [95% CI 2.687–6.947]; P < 0.001); increased fasting plasma glucose (FPG) (AOR 2.319 [95% CI 1.564–3.438]; P < 0.001); elevated D-dimer (AOR 2.117 [95% CI 1.407–3.184]; P < 0.001); elevated fibrinogen (AOR 1.601 [95% CI 1.077–2.380]; P = 0.20); and reduced estimated glomerular filtration rate (eGFR) (AOR 2.279 [95% CI 1.519–3.419]; P < 0.001).

**Conclusions:**

Our findings demonstrated that elevated ALT was independently associated with increased in-hospital all-cause mortality in patients with AMI. Other risk factors were increased age, FPG, D-dimer, and fibrinogen and decreased eGFR.

## Introduction

Coronary atherosclerotic disease (CAD) is the main source of cardiovascular disease (CVD), causing more than 7 million deaths worldwide each year. In recent decades, there have been substantial declines in cardiovascular disease mortality across much of Europe [1]. The latest guidelines from the European Heart Association, published in 2019, indicate that CAD is a pathologic process characterized by atherosclerotic plaque accumulation in the epicardial arteries, which is either obstructive or non-obstructive [2]. Acute coronary syndrome (ACS) refers to an acute ischemic syndrome of the heart caused by new thrombosis secondary to rupture or erosion of an unstable coronary artery atherosclerotic plaque. It includes unstable angina and acute myocardial infarction (AMI), either ST-segment elevation myocardial infarction (STEMI) or non-STEMI (NSTEMI).

AMI is associated with high morbidity and mortality. Mortality is influenced by many factors, including advanced age, smoking status, and the presence of hypertension, diabetes mellitus (DM), dyslipidemia, or obesity. Elevated levels of liver transaminases, specifically alanine aminotransferase (ALT) and aspartate aminotransferase (AST), are used as markers of hepatic dysfunction and have attracted attention as emerging risk factors for CVD.

Recent studies have investigated the role of liver transaminases as independent predictors of cardiac-related morbidity and mortality [3, 4]. Several prospective epidemiologic studies have suggested that hepatic dysfunction is common in cardiac disease [5, 6]. When no other causes of liver injury are identified, elevations of liver aminotransferases are associated with an elevated risk of cardiac-related mortality [7]. The goal of this study was to evaluate the association between elevated liver transaminases and in-hospital all-cause mortality in patients with AMI.

## Methods

### Study population

**Fig. 1:**
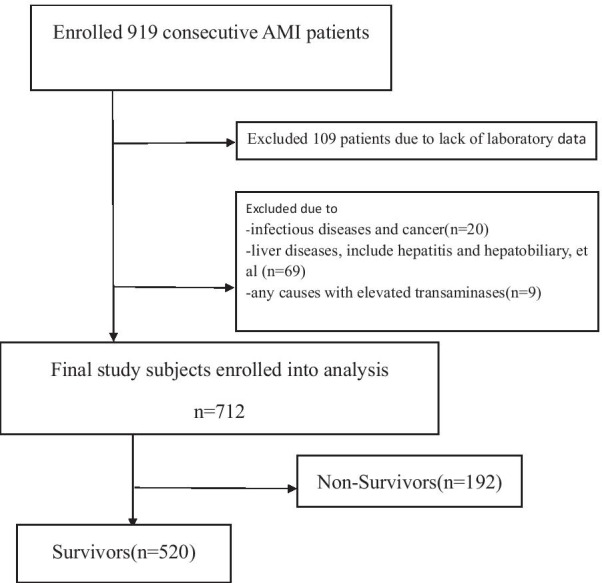
Flowchart of the enrolled patients

A population-based retrospective case–control study was conducted in the First Hospital of Jilin University, Changchun City, Jilin Province. General characteristics and routine laboratory tests of patients diagnosed with AMI were screened to identify patients for inclusion in the study. A total of 712 patients were included in the study (Fig. 1). Baseline demographic data, medical history, laboratory data, and clinical data during hospitalization were retrieved from our departmental heart disease electronic database (Table 1) and all methods were performed in accordance with the relevant guidelines and regulations.

**Table 1:** Clinical characteristics

Characteristics	NSTEMI (n = 238)	STEMI (n = 474)	P value
Age (y)	68.00 (60.00, 78.25)	65.00 (56.00, 73.00)	< 0.001
Male, n (%)	153 (64.3)	327 (69.0)	0.207
History of drinking alcohol, n (%)	67 (28.2)	133 (28.1)	0.979
Diabetes mellitus, n (%)	81 (34.0)	135 (28.5)	0.128
Hypertension, n (%)	144 (60.5)	247 (52.1)	0.034
History of smoking, n (%)	138 (58.0)	270 (57.0)	0.795
Family history of CVD, n (%)	7 (2.9)	13 (2.7)	0.880
Prior cerebral infarction, n (%)	27 (11.3)	72 (15.2)	0.162
Prior MI, n (%)	52 (21.8)	47 (9.9)	< 0.001
WBC count (10^9^/L)	9.10 (6.99, 11.89)	10.52 (8.42, 13.52)	< 0.001
Neutrophil count (10^9^/L)	6.68 (4.85, 9.27)	8.28 (6.34, 11.29)	< 0.001
RBC count (10^12^/L)	4.50 (3.98, 4.92)	4.62 (4.24, 5.01)	0.007
Hemoglobin (g/L)	136.50 (120.00, 153.00)	142.00 (127.00, 155.00)	0.005
Platelet count (10^9^/L)	218.50 (184.75, 257.25)	225.00 (187.50, 264.00)	0.314
FPG (mmol/L)	6.04 (4.93, 8.37)	6.02 (5.04, 8.82)	0.831
Triglycerides (mmol/L)	1.46 (1.05, 1.94)	1.44 (0.98, 2.17)	0.953
TC (mmol/L)	4.68 (3.83, 5.41)	4.62 (3.93, 5.33)	0.917
HDL-C (mmol/L)	1.14 (0.94, 1.38)	1.14 (0.96, 1.34)	0.884
LDL-C (mmol/L)	2.84 (2.20, 3.38)	2.83 (2.29, 3.39)	0.707
AST (U/L)	49.90 (29.95, 122.48)	137.10 (53.90, 252.08)	< 0.001
ALT (U/L)	27.55 (18.35, 51.83)	44.15 (27.20, 72.43)	< 0.001
GGT (U/L)	31.70 (21.00, 58.95)	36.75 (23.48, 59.15)	0.146
ALP (U/L)	89.60 (73.03, 106.43)	87.7 0(73.30, 106.43)	0.663
Cholinesterase (U/L)	7907.50 (6330.00, 9412.50)	8072.50 (6471.50, 9471.00)	0.394
Albumin (g/L)	36.85 (34.28, 40.10)	36.80 (34.10, 39.23)	0.521
T-Bil (µmol/L)	13.70 (9.88, 18.40)	13.50 (9.78, 18.70)	0.931
D-Bil (µmol/L)	4.00 (2.80, 5.00)	4.00 (3.00, 5.30)	0.864
PTA (%)	101.00 (93.00, 111.00)	101.00 (92.00, 109.00)	0.319
PT (s)	11.30 (10.70, 11.73)	11.30 (10.80, 11.90)	0.339
INR	0.97 (0.92, 1.01)	0.97 (0.92, 1.02)	0.318
Fibrinogen (g/L)	3.46 (3.05, 4.15)	3.51 (2.94, 4.21)	0.942
Tn I (ng/mL)	0.79 (0.15, 5.01)	5.47 (0.43, 20.75)	< 0.001
Myoglobin (ng/mL)	239.50 (109.00, 500.00)	500.00 (173.75, 500.00)	< 0.001
CK-MB (ng/mL)	6.60 (2.30, 25.58)	24.85 (4.38, 67.90)	< 0.001
BNP (pg/mL)	175.00 (43.93, 1020.00)	119.50 (22.88, 489.25)	0.005
D-dimer (ng/mL)	204.00 (100.00, 702.00)	170.00 (100.00, 749.25)	0.564
Creatinine (µmol/L)	76.35 (63.38, 105.80)	72.05 (60.30, 89.28)	0.007
BUN (mmol/L)	6.61 (5.17, 8.95)	6.04 (5.09, 7.62)	0.006
Liver-protecting, n (%)	16 (6.7)	61 (12.9)	0.013
In-hospital death, n (%)	72 (30.3)	120 (25.3)	0.162

Continuous variables are expressed as median (25th, 75th percentiles). Categorical variables are shown as numbers (percentages)

ALP, alkaline phosphatase; ALT, alanine aminotransferase; AST, aspartate aminotransferase; BNP, brain natriuretic peptide; BUN, blood urea nitrogen; CK-MB, creatine kinase MB; CVD, cardiovascular disease; D-Bil, direct bilirubin; FPG, fasting plasma glucose; GGT, γ-glutamyl transpeptidase; HDL-C, high-density lipoprotein cholesterol; INR, international normalized ratio; LDL-C, low-density lipoprotein cholesterol; MI, myocardial infarction; NSTEMI, non–ST-segment elevation myocardial infarction; PT, prothrombin time; PTA, prothrombin time activity; RBC, red blood cell; STEMI, ST-segment elevation myocardial infarction; T-Bil, total bilirubin; TC, total cholesterol; Tn I, cardiac troponin I; WBC, white blood cell

## Definitions

The diagnostic criteria for AMI were in accordance with the 2017 guidelines from the European Society of Cardiology (ESC) [8]. Myocardial injury was defined as at least one cardiac troponin value above the 99th percentile upper reference limit. STEMI was defined as persistent chest discomfort or other symptoms suggestive of ischemia and ST-segment elevation in at least two contiguous leads. Ischemic symptoms in the absence of ST-segment elevation at presentation were designated as NSTEMI [8]. Hypoxic liver injury (HLI) was defined as at least one serum transaminase level more than twice the upper limit of normal (ULN). The ULN for ALT was defined as 50 U/L for men and 40 U/L for women, whereas the ULN for AST was defined as 40 U/L for men and 35 U/L for women. The World Health Organization (WHO) defines impaired fasting glucose as a fasting plasma glucose (FPG) value of 6.1 mmol/L or higher, whereas the American Diabetes Association recommends a cutoff value of 5.6 mmol/L [9]. In this study, we used the WHO cutoff value. Estimated glomerular filtration rate (eGFR) was calculated as 175 × plasma creatinine^–1.234^ × age^–0.179^ × 0.79 if female [10]. Elevated D-dimer level was defined as a serum concentration ≥ 400 ng/mL and increased fibrinogen was defined as a serum concentration ≥ 3.5 g/L.

### Exclusion criteria

Patients with incomplete data were excluded from the study. We also excluded patients with elevated transaminases for various non-AMI reasons, including hepatitis and hepatic cirrhosis, chronic schistosomiasis, hepatobiliary obstructive disease, bone disease, pancreatitis; cardiomyopathy, severe heart failure, severe renal insufficiency, and infectious diseases. It is well known that hyperlipidemia and fatty liver disease are risk factors for CVD. Patients diagnosed with fatty liver disease were not excluded from our study, but we did exclude patients with liver dysfunction attributed to fatty liver disease.

### Laboratory data

Peripheral blood samples obtained at the time of admission were tested for the following: complete blood count, creatinine, cardiac troponin I (Tn I), creatine kinase MB (CK-MB), myoglobin, potassium, sodium, brain natriuretic peptide (BNP), prothrombin time (PT), PT activity (PTA), fibrinogen, and D-dimer. Total cholesterol (TC), triglycerides, high-density lipoprotein cholesterol (HDL-C), low-density lipoprotein cholesterol (LDL-C), FPG, ALT, AST, alkaline phosphatase (ALP), γ-glutamyl transpeptidase (GGT), cholinesterase, albumin, total bilirubin (T-Bil), and direct bilirubin (D-Bil) were measured after 12 h of fasting following admission. All blood samples obtained at the time of admission were analyzed in the certified laboratory department of First Hospital of Jilin University.

### Statistical analysis

All data analysis was performed using SPSS version 22.0, and P < 0.05 indicated statistical significance. Continuous variables were represented by the median (25th and 75th percentiles) or mean ± standard deviation. Categorical data were described by counts and percentages. Continuous variables were compared using two-tailed independent sample t-tests, and categorical variables were compared using the chi-square test. Multivariate logistic regression models were used to identify risk factors for mortality in patients with AMI. These models included adjustments for potential confounding variables, and adjusted odds ratios (AORs) and 95% confidence intervals (CIs) were calculated. The logistic refression models were calibrated by Hosmer–Lemeshow test.

## Results

### Demographic and clinical characteristics

Baseline demographic and clinical characteristics of the 714 patients included in the study are shown in Table 1. Of the 474 patients with STEMI, 327 were male, 147 were female, and the median age was 65.0 years. In this group, 270 patients (57.0%) had a smoking history, 133 (28.1%) had a history of drinking alcohol, 247 (52.1%) had hypertension, 135 (28.5%) had DM, 47 (9.9%) had a prior myocardial infarction, and 72 (15.2%) had a prior cerebral infarction. Of the 238 patients with NSTEMI, 153 were male and 85 were female, with a median age of 67.0 years. In this group, 138 patients (58.0%) had a smoking history, 67 (28.2%) had a history of drinking alcohol, 144 (60.5%) had hypertension, 81 (34.0%) had DM, 52 (21.8%) had a previous myocardial infarction, and 27 (11.3%) had a prior cerebral infarction.

Patients with NSTEMI were older and more likely to have DM than those in the STEMI group. AST, ALT, white blood cell count, neutrophil count, Tn I, myoglobin, CK-MB, and in-hospital mortality were also higher in the STEMI group than in the NSTEMI group.

### Factors associated with in-hospital mortality in all patients

Multivariate logistic regression analysis was used to identify risk factors for in-hospital mortality in all 712 patients with AMI (Table 2). Age, sex, DM, smoking history, FPG, TC, triglycerides, AST, ALT, GGT, Tn I, D-dimer, fibrinogen, and eGFR were included in the multivariate analysis. Study participants with an ALT ≥ 2 ULN had an AOR of 2.240 (95% CI 1.331–3.771; P = 0.002), when compared with patients with an ALT < 2 ULN. Other factors associated with an increased odds of in-hospital mortality were age ≥ 65 years (AOR 4.320 [95% CI 2.687–6.947]; P < 0.001); FPG (AOR 2.319 [95% CI 1.564–3.438]; P < 0.001); decreased eGFR (AOR 2.279 [95% CI 1.519–3.419]; P < 0.001); elevated fibrinogen (AOR 1.601 [95% CI 1.077–2.380]; P = 0.020); and elevated D-dimer (AOR 2.117 [95% CI 1.407–3.184]; P < 0.001).

**Table 2:** Multivariate analysis of variables associated with in-hospital mortality in all study patients

Variable	Survivors (n = 520)	Non-survivors (n = 192)	P value	AOR (95% CI)
Age (y)			< 0.001	4.320 (2.687–6.947)
< 65, n (%)	283 (54.4)	31 (16.1)		
≥ 65, n (%)	237 (45.6)	161 (83.9)		
Sex			0.298	0.795 (0.517–1.224)
Female, n (%)	149 (28.7)	83 (43.2)		
Male, n (%)	371 (71.3)	109 (56.8)		
DM			0.229	1.317 (0.841–2.063)
No, n (%)	387 (74.4)	109 (56.8)		
Yes, n (%)	133 (25.6)	83(43.2)		
History of smoking			0.364	1.317 (0.798–1.853)
No, n (%)	208 (40.0)	96 (50.0)		
Yes, n (%)	312 (60.0)	96 (50.0)		
FPG (mmol/L)			< 0.001	2.319 (1.564–3.438)
< 6.1, n (%)	302 (58.1)	65 (33.9)		
≥ 6.1, n (%)	218 (41.9)	127 (66.1)		
Triglycerides (mmol/L)			0.184	0.739 (0.472–1.155)
< 1.8, n (%)	326 (62.7)	142 (74.0)		
≥ 1.8, n (%)	194 (37.3)	50 (26.0)		
TC (mmol/L)			0.689	1.194 (0.502–2.843)
< 6.6, n (%)	490 (94.2)	180 (93.7)		
≥ 6.6, n (%)	30 (5.8)	12 (6.3)		
AST (U/L)			0.764	1.075 (0.671–1.722)
< 2 ULN, n (%)	237 (45.6)	79 (41.1)		
≥ 2 ULN, n (%)	283 (54.4)	113 (58.9)		
ALT (U/L)			0.002	2.240 (1.331–3.771)
< 2 ULN, n (%)	432 (83.1)	140 (72.9)		
≥ 2 ULN, n (%)	88 (16.9)	52 (27.1)		
GGT (U/L)			0.643	1.161 (0.618–2.182)
< 2 ULN, n (%)	468 (90.0)	163 (84.9)		
≥ 2 ULN, n (%)	52 (10.0)	29 (15.1)		
D-dimer (ng/mL)			< 0.001	2.117 (1.407–3.184)
< 400, n (%)	374 (71.9)	67 (34.9)		
≥ 400, n (%)	146 (28.1)	125 (65.1)		
Tn I (ng/mL)			0.736	0.929 (0.604–1.429)
< 2.5, n (%)	266 (51.2)	87 (45.3)		
≥ 2.5, n (%)	254 (48.8)	105 (54.7)		
Fibrinogen (g/L)			0.020	1.601 (1.077–2.380)
< 3.5, n (%)	289 (55.6)	73 (38.0)		
≥ 3.5, n (%)	231 (44.4)	119 (62.0)		
eGFR (mL/min/1.73 m^2^)			< 0.001	2.279 (1.519–3.419)
≥ 90, n (%)	373 (71.7)	65 (33.9)		
< 90, n (%)	147 (28.3)	127 (66.1)		

Age, sex, DM, smoking history, FPG, TC, triglycerides, AST, ALT, GGT, Tn I, D-dimer, fibrinogen, and eGFR were included in the multivariate logistic regression analysis

ALT, alanine aminotransferase; AOR, adjusted odds ratio; AST, aspartate aminotransferase; CI, confidence interval; DM, diabetes mellitus; eGFR, estimated glomerular filtration rate; FPG, fasting plasma glucose; GGT, γ-glutamyl transpeptidase; NSTEMI, non–ST-segment elevation myocardial infarction; STEMI, ST-segment elevation myocardial infarction; T-Bil, total bilirubin; TC, total cholesterol; Tn I, cardiac troponin I; ULN, upper limit of normal

**Fig. 2:**
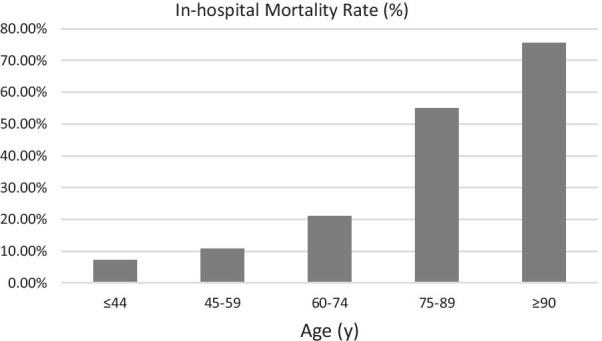
In-hospital mortality rates of patients with acute myocardial infarction categorized by age at presentation. There were 42, 167, 322, 167, and 14 patients in the ≤ 44 year, 45–59 year, 60–74 year, 75–89 year, and ≥ 90 year age groups, respectively. The corresponding mortality rates for each group were 7.1%, 10.8%, 21.1%, 55.1%, and 75.6%

**Fig. 3:**
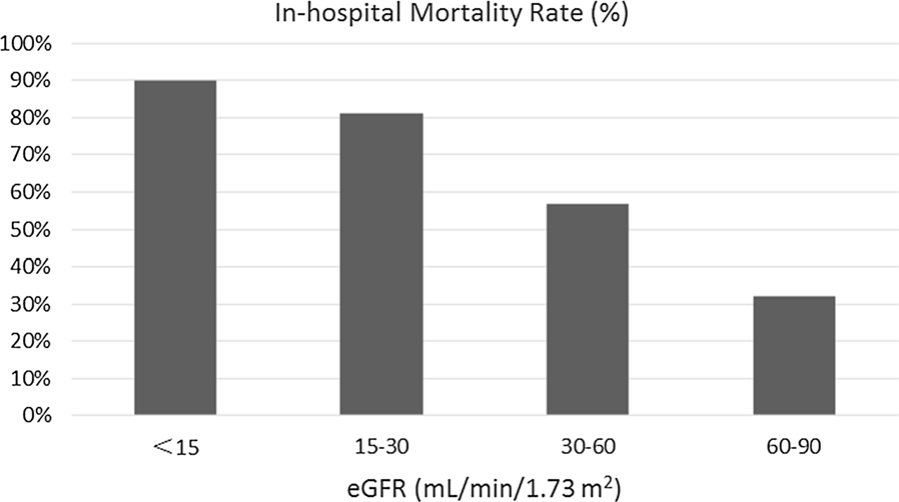
In-hospital mortality rates of patients with acute myocardial infarction categorized by estimated glomerular filtration rate (eGFR) at presentation. There were 11, 25, 88, and 150 patients in the < 15, 15–30, 30–60, and 60–90 mL/min/17.3 m^2^ eGFR groups, respectively. The corresponding mortality rates for each group were 90.0%, 81.0%, 56.8%, and 32.0%

**Fig. 4:**
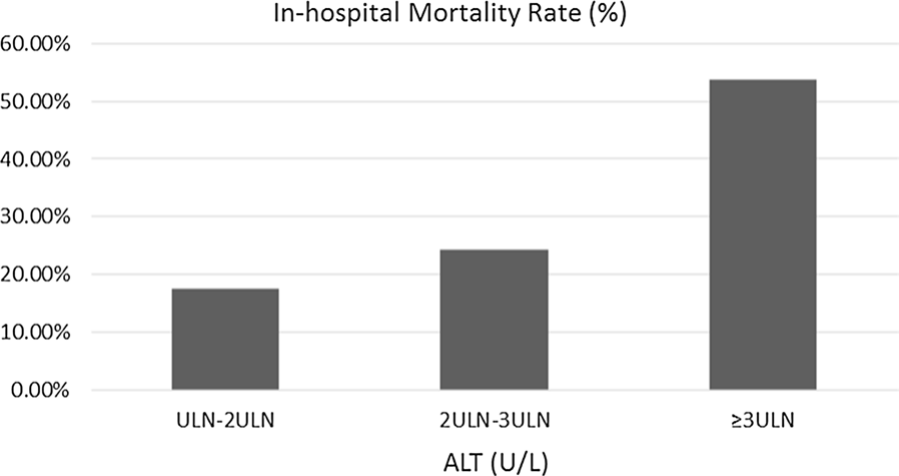
In-hospital mortality rates of patients with acute myocardial infarction categorized by alanine aminotransferase (ALT) concentration at presentation. There were 212, 70, and 65 patients in the upper limit of normal (ULN)–2 ULN, 2 ULN–3 ULN, and ≥ 3 ULN ALT groups, respectively. The corresponding mortality rates for each group were 17.5%, 24.3%, and 53.8%

Figures 2, 3 and 4 show the differences in mortality rates according to age, ALT, and eGFR subgroups for the entire study population. These figures clearly illustrate the increase in mortality with increasing age and ALT and decreasing eGFR.

### Factors associated with in-hospital mortality according to type of myocardial infarction

Multivariate logistic regression analysis was used to identify risk factors for all-cause in-hospital death in 474 patients with STEMI and 238 patients with NSTEMI (Tables 3 and 4). Factors included in the multivariate analysis were the same as those included when analyzing all study participants. Patients with an ALT ≥ 2 ULN had a higher risk of in-hospital mortality in both the STEMI and NSTEMI groups (AOR 1.915 [95% CI 1.028–3.565] and AOR 3.698 [95% CI 1.302–10.500], respectively; P < 0.001 for both). Other factors associated in-hospital mortality in both the STEMI and NSTEMI groups were age ≥ 65 year (AOR 4.668 [95% CI 2.684–8.117] and AOR 2.753 [95% CI 1.053–7.199], respectively); FPG (AOR 2.010 [95% CI 1.236–3.267] and AOR 3.823 [95% CI 1.820–7.808], respectively; P < 0.001 for both); and elevated D-dimer (AOR 1.937 [95% CI 1.175–3.194] and AOR 3.053 [95% CI 1.471–6.334], respectively). Elevated fibrinogen was associated with an increased odds of in-hospital mortality in patients with STEMI (AOR 1.810 [95% CI 1.113–2.944]; P = 0.017), but not in patients with NSTEMI.

**Table 3:** Multivariate analysis of variables associated with in-hospital mortality in patients with STEMI

Variables	Survivors (n = 35)	Non-survivors (n = 120)	P value	AOR (95% CI)
Age ≥ 65 year, n (%)	151 (42.7)	97 (80.8)	< 0.001	4.668 (2.684–8.117)
Male, n (%)	256 (72.3)	71(59.2)	0.542	0.848 (0.498–1.441)
DM, n (%)	64 (25.4)	45 (37.5)	0.610	1.161 (0.654–2.062)
Smoking, n (%)	212 (59.9)	58 (48.3)	0.864	1.046 (0.627–1.743)
FPG ≥ 6.1 mmol/L, n (%)	154 (43.5)	76 (63.3)	0.005	2.010 (1.236–3.267)
TG ≥ 1.8 mmol/L, n (%)	134 (37.9)	31 (25.8)	0.208	0.699 (0.400–1.221)
TC ≥ 6.6 mmol/L, n (%)	19 (5.4)	5 (4.2)	0.930	0.948 (0.288–3.117)
AST ≥ 2 ULN, n (%)	230 (65.0)	76 (63.3)	0.529	0.832 (0.470–1.474)
ALT ≥ 2 ULN, n (%)	74 (15.6)	27 (22.5)	0.041	1.915 (1.028–3.565)
GGT ≥ 2 ULN, n (%)	35 (0.9)	18 (15.0)	0.287	1.502 (0.710–3.178)
DD ≥ 400 ng/mL, n (%)	103 (29.1)	75 (62.5)	0.010	1.937 (1.170–3.194)
Tn I ≥ 2.5 ng/mL, n (%)	201 (56.8)	77 (64.2)	0.952	0.984 (0.572–1.692)
Fib ≥ 3.5 g/L, n (%)	163 (46.0)	76 (63.3)	0.017	1.810 (1.113–2.944)
eGFR (mL/min/1.73 m^2^) < 90, n (%)	95 (26.8)	70 (58.3)	0.017	1.834 (1.112–3.023)

Age, sex, DM, smoking history, FPG, TC, triglycerides, AST, ALT, GGT, Tn I, D-dimer, fibrinogen, and eGFR were included in the multivariate logistic regression analysis

ALT, alanine aminotransferase; AOR, adjusted odds ratio; AST, aspartate aminotransferase; CI, confidence interval; DM, diabetes mellitus; eGFR, estimated glomerular filtration rate; FPG, fasting plasma glucose; GGT, γ-glutamyl transpeptidase; NSTEMI, non–ST-segment elevation myocardial infarction; STEMI, ST-segment elevation myocardial infarction; T-Bil, total bilirubin; TC, total cholesterol; Tn I, cardiac troponin I; ULN, upper limit of normal

**Table 4:** Multivariate analysis of variables associated with in-hospital mortality in patients with NSTEMI

Variables	Survivors (n = 166)	Non-survivors (n = 72)	P value	AOR (95% CI)
Age ≥ 65y, n (%)	86 (51.8)	64 (88.9)	0.039	2.753 (1.053–7.199)
Male, n (%)	115 (69.3)	38 (52.8)	0.225	0.604 (0.267–1.365)
DM, n (%)	43 (25.9)	38 (52.8)	0.163	1.786 (0.790–4.038)
Smoking, n (%)	100 (60.2)	38 (52.8)	0.123	1.920 (0.838–4.403)
FPG ≥ 6.1 mmol/L, n (%)	64 (38.6)	51 (70.8)	< 0.001	3.823 (1.820–7.808)
TG ≥ 1.8 mmol/L, n (%)	60 (36.1)	19 (26.4)	0.886	1.065 (0.452–2.506)
TC ≥ 6.6 mmol/L, n (%)	11 (6.6)	7 (9.7)	0.617	1.431 (0.351–5.835)
AST ≥ 2ULN, n (%)	53 (31.9)	37 (51.4)	0.277	1.681 (0.659–4.286)
ALT ≥ 2 ULN, n (%)	13 (7.8)	19 (26.4)	0.014	3.698 (1.302–10.500)
GGT ≥ 2 ULN, n (%)	17 (10.2)	11 (15.3)	0.076	0.280 (0.069–1.143)
DD ≥ 400 ng/mL, n (%)	43 (25.9)	50 (69.4)	0.003	3.053 (0.471–6.334)
Tn I ≥ 2.5 ng/mL, n (%)	53 (31.9)	28 (38.9)	0.712	0.854 (0.371–1.969)
Fib ≥ 3.5 g/L, n (%)	68 (41.0)	43 (59.7)	0.612	1.210 (1.578–2.535)
eGFR < 90 mL/min/1.73 m^2^, n (%)	52 (31.3)	57 (79.2)	< 0.001	4.063 (1.890–8.737)

Age, sex, DM, smoking history, FPG, TC, triglycerides, AST, ALT, GGT, Tn I, D-dimer, fibrinogen, and eGFR were included in the multivariate logistic regression analysis

ALT, alanine aminotransferase; AOR, adjusted odds ratio; AST, aspartate aminotransferase; CI, confidence interval; DM, diabetes mellitus; eGFR, estimated glomerular filtration rate; FPG, fasting plasma glucose; GGT, γ-glutamyl transpeptidase; NSTEMI, non–ST-segment elevation myocardial infarction; STEMI, ST-segment elevation myocardial infarction; T-Bil, total bilirubin; TC, total cholesterol; Tn I, cardiac troponin I; Fib, fibrinogen; TG, triglycerides; DD, D-dimer; ULN, upper limit of normal

## Discussion

One of the most important factors associated with an increased risk of in-hospital mortality in patients with an AMI was an elevated ALT. The liver is a vital organ that is very sensitive to hemodynamic changes because of its complex vascular system and high metabolic activity [11]. Every 10-mm Hg decrease in arterial blood pressure decreases hepatic arterial blood flow by approximately 10% [12]; therefore, abnormal serum transaminases are often observed in patients with AMI. A few recent studies reported that elevated serum transaminases were independently associated with poor clinical outcomes in patients with AMI. The extent of liver injury, in particular in patients with preexisting metabolic syndrome, may have a direct impact on cardiac outcomes [13].

Hypoxic liver injury (HLI) is commonly diagnosed in the emergency room in patients with STEMI (in 22% of patients), and those patients with HLI have higher mortality rate and higher frequency of major adverse cardiovascular events (MACE) after percutaneous coronary intervention [14]. Using multivariable regression analysis, Jantti et al. reported that a > 20% increase in ALT was associated with an increased risk of 90-day mortality independent of other known risk factors [15]. However, the association between liver transaminases and CVD remains a subject of debate. Authors of a narrative review based on prospective data concluded that the available evidence did not support a linear relationship between ALT and CVD events [16]. Differences in the effects of specific aminotransferases have not been clearly elucidated and require further research.

A few potential mechanisms have been proposed to explain the association between elevated ALT levels and increased in-hospital all-cause mortality in patients with AMI. For example, the liver has high metabolic activity and perfusion rates, and acute circulatory changes, such as AMI-related hypotension, will directly influence hepatic blood flow and thereby increase ALT and AST. Liver transaminases may also be elevated because of hepatic congestion from acute right ventricular dysfunction. Current research suggests that venous congestion, reduced capability of hepatocytes to extract oxygen, and reperfusion injury are of particular importance [12]. Although the source of elevated serum transaminases might be ischemic myocardial tissue, increased levels of these enzymes often reflect HLI secondary to both impaired forward perfusion and passive backward congestion, which are prevalent during STEMI [6]. In addition, an analysis of asymptomatic individuals found that increased levels of serum ALT (even high-normal levels) were associated with markers of CVD [17]. It is known that incomplete peripheral lipolysis of very low–density lipoprotein (VLDL) particles over-enriched with triglycerides increases the number of small dense LDL (sdLDL) particles, which carry a disproportionately large amount of LDL-C [17–19]. ALT levels are directly related to desmosterol: TC ratio, a marker of cholesterol synthetic activity [20], thereby suggesting that ALT elevations are associated with increased hepatic triglyceride and cholesterol output in VLDL particles and lead to increased production of sdLDL [17].

In this study, we found that increased patient age was also significantly associated with mortality, which is consistent with previous reports of higher mortality in elderly patients with AMI [21–23]. In the CADILLAC trial, both cardiac and noncardiac causes of death were more frequent in older patients [21]. Myocytes are continuously lost as the heart ages, and senescent cells exhibit increased susceptibility to microvasculature reperfusion injury. D-dimer, one of the degradation products of fibrin, is produced when fibrinolytic enzymes degrade fibrin clots produced by thrombin. Choi et al. reported that in STEMI patients undergoing primary percutaneous coronary intervention, high D-dimer levels on admission were associated with a larger myocardial infarction size [24]. In the current study, higher D-dimer levels were associated with higher all-cause in-hospital mortality in patients with AMI; this finding is consistent with the results reported by Kikkert et al. [25].

In the current study, another important predictor of early mortality in patients with AMI was reduced eGFR. A previous study reported that among patients with AMI, any degree of preexisting renal impairment should be considered a potent, independent, and easily identifiable risk factor for cardiovascular complications, with each 10-unit reduction in eGFR being associated with a 10% increased risk for death and nonfatal cardiovascular outcomes [26]. In general, patients with chronic kidney disease have more cardiovascular risk factors and a higher incidence of MACE with AMI and acute kidney injury (AKI) was revealed to be an independent long-term prognostic indicator in patients with STEMI [27]. Even small, subclinical elevations of serum creatinine that do not fulfil the consensus criteria for acute kidney injury have been independently associated with a twofold increased risk of adverse in-hospital outcomes after AMI [28]. Our findings suggested that serum eGFR was an independent predictor of in-hospital all-cause mortality after AMI, with mortality increasing as eGFR decreased. Various factors associated with impaired renal function, such as insulin resistance, oxidative stress, inflammation, endothelial dysfunction, vascular calcifications, and hypercoagulability, may contribute to adverse outcomes in patients with ACS [29].

The 2019 ESC guidelines regarding diabetes, pre-diabetes, and cardiovascular diseases developed in collaboration with the European Association for the Study of Diabetes concluded that patients with DM and established CVD have very high cardiovascular risk [30]. Our findings that elevated FPG, but not DM, was associated with mortality suggest that glycemic control is the critical determinant of the risk of death. Previous studies have shown that severe hyperglycemia on admission was associated with increased in-hospital mortality in AMI patients with or without DM [31, 32]. Hyperglycemia can induce abnormalities in endothelial and vascular smooth muscle cell function, as well as a propensity to thrombosis, thereby contributing to atherosclerosis and its complications [33], as well as hypercoagulability and impaired fibrinolysis [34].

This study had several limitations. As a retrospective study, it relied on the accuracy of data in the medical records. It was also based on data from a single center, whose population consisted primarily of a single ethnic group of individuals located in Northeast China. Because of the climate, diet, and other factors, the incidence of cardiovascular disease in this region is significantly higher than in other regions, and the data may not reflect a general population of patients with AMI. Furthermore, although we excluded patients diagnosed with and treated for various liver diseases (including hepatobiliary malignancies, liver fibrosis and cirrhosis, and viral hepatitis) based on medical records, some factors potentially affecting transaminase levels, such as undiagnosed liver disease or medications affecting liver function, may have remained undetected and therefore not been considered when excluding patients.

## Conclusions

This retrospective study focused on non-invasive markers of in-hospital death from any cause after AMI. Increased risk of in-hospital all-cause mortality was significantly associated with increased levels of ALT, older age, FPG, D-dimer, and fibrinogen and decreased eGFR. Our results therefore suggest that elevation of the serum transaminase ALT, which likely reflects HLI, may be a useful marker of increased risk of early mortality in patients with AMI.

## Data Availability

The datasets used and analyzed during the current study are available from the corresponding author on request.

## Abbreviations

ALP: Alkaline phosphatase
ALT: Alanine aminotransferase;
AST: Aspartate aminotransferase
BNP: Brain natriuretic peptide
BUN: Blood urea nitrogen
CK-MB: Creatine kinase MB
CVD: Cardiovascular disease
D-Bil: Direct bilirubin
FPG: Fasting plasma glucose
GGT: γ-Glutamyl transpeptidase
HDL-C: High-density lipoprotein cholesterol
INR: International normalized ratio
LDL-C: Low-density lipoprotein cholesterol
MI: Myocardial infarction
NSTEMI: Non–ST-segment elevation myocardial infarction
PT: Prothrombin time
PTA: Prothrombin time activity
RBC: Red blood cell
STEMI: ST-segment elevation myocardial infarction
T-Bil: Total bilirubin
TC: Total cholesterol
Tn I: Cardiac troponin I
WBC: White blood cell
AOR: Adjusted odds ratio
CI: Confidence interval
eGFR: Estimated glomerular filtration rate
CAD: Coronary atherosclerotic disease
DM: Diabetes mellitus
HLI: Hypoxic liver injury
MACE: Major adverse cardiovascular events
ESC: European Society of Cardiology
WHO: World Health Organization
